# Bilayer C_60_ Polymer/*h*-BN Heterostructures: A DFT Study of Electronic and Optic Properties

**DOI:** 10.3390/polym16111580

**Published:** 2024-06-03

**Authors:** Leonid A. Chernozatonskii, Aleksey I. Kochaev

**Affiliations:** 1 Emanuel Institute of Biochemical Physics RAS, 4 Kosygin Street, 119334 Moscow, Russia; 2 Scientific School on Chemistry and Technology of Polymer Materials, Plekhanov Russian University of Economics, 117997 Moscow, Russia; 3 Research and Education Center “Silicon and Carbon Nanotechnologies”, Ulyanovsk State University, 42 Leo Tolstoy Street, 432017 Ulyanovsk, Russia; 4 Laboratory of 2D Nanomaterials in Electronics, Photonics and Spintronics, National Research Nuclear University “MEPhI”, 31 Kashirskoe sh., 115409 Moscow, Russia

**Keywords:** fullerenes, bilayers, boron nitride, graphene, van der Waals heterostructures, DFT

## Abstract

Interest in fullerene-based polymer structures has renewed due to the development of synthesis technologies using thin C_60_ polymers. Fullerene networks are good semiconductors. In this paper, heterostructure complexes composed of C_60_ polymer networks on atomically thin dielectric substrates are modeled. Small tensile and compressive deformations make it possible to ensure appropriate placement of monolayer boron nitride with fullerene networks. The choice of a piezoelectric boron nitride substrate was dictated by interest in their applicability in mechanoelectric, photoelectronic, and electro-optical devices with the ability to control their properties. The results we obtained show that C_60_ polymer/h-BN heterostructures are stable compounds. The van der Waals interaction that arises between them affects their electronic and optical properties.

## 1. Introduction

Recent advances in nanoelectronics, hydrogen energy, and biotechnology are closely related to new nanoscale materials and structures with unique physical properties. Examples of such objects are fullerenes [1,2,3]. These carbon macromolecules, in particular C_60_ fullerene, are used in nonlinear optics, semiconductor technology, electric batteries, pharmacology, the chemical industry, and biomedical technologies [4]. The discovery of the synthesis of monolayer C_60_ polymers has returned interest in fullerene polymer structures [5]. They have been shown to be good semiconductors with anisotropic optical properties. Quantum chemical modeling confirms the stability of polymerized fullerenes and proves that they have interesting anisotropic optical, mechanical, and thermoelectric characteristics [6]. A high degree of hydrogen adsorption of fullerene layers placed on a graphene substrate is also shown [7].

At the same time, dielectric substrates are of great importance in nanoengineering. The most famous and easily reproducible is a substrate made of single or multilayer boron nitride [8]. As far as we know, there has been no study of the placement of fullerenes on a single-layer boron nitride substrate, nor has there been a study of sandwiched polymerized fullerenes between two layers of boron nitride. In this paper, we fill these gaps by focusing on the structural, electronic, and optical properties of heterostructures formed by polymerized fullerenes and boron nitride layers. We considered two types of structures, taking into account the stability of polymer monolayers, namely the quasi-hexagonal fullerene phase qhPC_60_, in which each fullerene is bonded to its neighbors through four single and two cycloaddition bonds [2 + 2], and the hexagonal fullerene phase hPC_60_, which is close to the qhPC_60_ structure in atomic structure (the 2D-18 polymer in the B. Mortazavi designation [9]), where the C_60_ molecule is bonded by six [2 + 2] bonds to its neighbors—a model previously considered one of the pressure-induced C_60_ polymers [10].

The stability of the considered fullerene networks is achievable by doping with magnesium and potassium [11]. It is expected that fullerene networks in heterostructures will be more stable [12,13,14]. In general, planar C_60_ polymers are of great interest [15]. The combined use of C_60_ polymer and *h*-BN monolayers has important practical benefits. Firstly, this heterostructure is a semiconductor material on a strong piezoelectric dielectric; therefore, it becomes possible to change the electronic spectrum (conductivity and optical response) using structural deformation and (or) doping with foreign atoms. These can be mechanical–electrical sensors that respond to the appearance of electrical voltage on the film or optically excited ones. Secondly, this hybrid material is easy to manufacture due to the currently well-studied components that make it up.

## 2. Computational Methods

Computational design and procedure were carried out using the Quantum ATK software 2021.06 [16]. Structural optimization and calculation of the main characteristics of heterostructures under consideration were performed using atomistic modeling based on density functional theory (DFT) in the linear combination of atomic orbitals (LCAOs) approximation. The generalized gradient approximation (GGA) for the exchange and correlation potential expressed by the Perdew−Burke−Ernzerhof (PBE) functional and the projector augmented-wave potential (PAW) are used to obtain equilibrium atomic configurations of the heterostructures [17]. To obtain the electronic spectra, the Heyd−Scuseria−Ernzerhof hybrid exchange-correlation functional is applied [18]. All calculations were carried out using periodic boundary conditions. To neglect the influence of the periodic images in the *z*-direction perpendicular to the sheet, we take the value of the cell parameter along *z* equal to 15 Å. The van der Waals interaction was taken into account using the DFT-D2 method of Grimme [19]. The use of these approaches has previously made it possible to predict entire families of two-dimensional materials [20].

The projected density of states (PDOSs) is the relative contribution of a particular atom (or orbital) to the total density of states. Associated with a given projection *M*, the PDOS is defined as

(1) $$D_{M}\left(\epsilon \right)=\sum_{n}\delta \left(\epsilon -\epsilon_{n}\right)\langle \varphi_{n}|\hat{P_{M}}|\varphi_{n}\rangle ,$$

where *φ_n_* are the eigenstates, $\left|\hat{P_{M}}\right|$ is a projection operator [21], and *n* includes all the quantum numbers of the system.

The optical properties were characterized by the absorption coefficient α_a_. Its calculation is based on solving a system consisting of the Kubo-Greenwood equation for the components of the susceptibility tensor *χ_ij_* [22]

(2) $$\chi_{\mathrm{ij}}\left(\omega \right)=-\frac{e^{2}\hbar^{4}}{m^{2}\varepsilon_{0}V\omega^{2}}\sum_{\mathrm{nm}}\frac{f\left(E_{m}\right)-f\left(E_{\overset{´}{m}}\right)}{E_{\overset{´}{m}m}-\hbar \omega -i\Gamma }\pi_{\overset{´}{m}m}^{i}\pi_{\overset{´}{\mathrm{mm}}}^{j},$$

and the coupling equations

$$n+i\kappa =\sqrt{\varepsilon_{r}},$$

(3) $$\varepsilon_{r}\left(\omega \right)=(1+\chi (\omega )),$$

$$\alpha_{a}=2\frac{\omega }{c}\kappa ,$$

where *V* is a volume of the considered cell, ω is a electromagnetic wave frequency, *f* is the Fermi-Dirac function, Γ = 0.1 eV is the broadening, $\pi_{\overset{´}{m}m}^{i}$ is the *i*-th dipole matrix element between the states *m′* and *m*, *n* is the refractive index, *κ* is the extinction coefficient, *ε_r_* is the relative dielectric constant, and *c* is the speed of light.

To clearly see the redistribution of electrons on the components of the considered heterostructures, we study the electron localization function (ELF). The ELF is defined as [23].

$$\mathrm{ELF}=\frac{1}{1+{\left(D/D_{h}\right)}^{2}},$$

(4) $$D=\frac{1}{2}\sum_{i}{\left|\nabla \varphi_{i}\right|}^{2}-\frac{1}{8}\frac{{\left|\nabla \rho \right|}^{2}}{\rho },$$

$$D_{h}=\frac{3}{10}{(3\pi^{2})}^{2/3}\rho^{5/3},$$

where *φ* is the Kohn–Sham orbital, and *ρ* is the local density. The electron localization function, ranging from 0 to 1, takes the value 0 if there are regions of space where the electrons are perfectly delocalized, and it takes a value of 1 if high electron localization can be observed.

## 3. Results and Discussion

### 3.1. Geometric Structures and Properties of Individual Units

**Figure 1:**
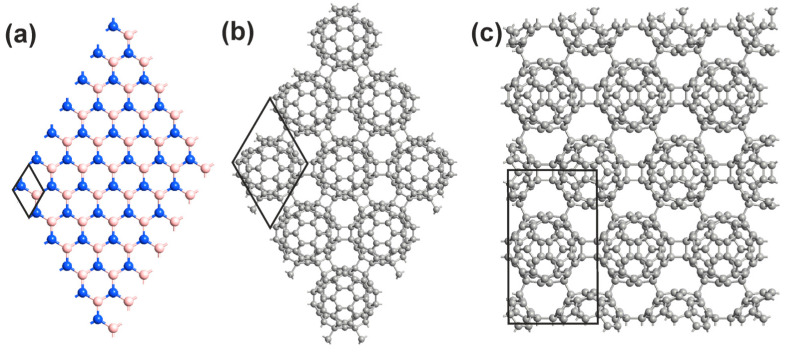
Atomic configurations and unit cells of optimized monolayer boron nitride (**a**) and two polymerized sets of fullerenes (**b**,**c**). Blue, pale pink, and grey colors denote the nitrogen, boron, and grey atoms. Black lines highlight the unit cells of the heterostructures. Lattice parameter is 2.50 Å for monolayer boron nitride; 9.21 Å for the HPC_60_ fullerene phase; and 15.8 Å and 9.17 Å for the qHPC_60_ fullerene phase.

Ways for incorporating C_60_ and other fullerene molecules into polymer structures are described in detail in [9,24]. We limited ourselves to the hexagonal (HPC_60_) and quasi-hexagonal fullerene phases (qHPC_60_). The two considered types are stable. The presented fullerene sets are held covalent bonds. To calculate the binding energy acting between individual C_60_ molecules in polymerized fullerene sets, we use the following expression

(5) $$E_{b}=E_{\mathrm{HPC}60/\mathrm{qHPC}60}\;-E_{C_{60}}$$

where *E_HPC60/qHPC60_* is the total energy of cells containing the atomic configuration of one polymerized fullerene phase, $E_{C_{60}}$ is the same, but for a free fullerene molecule. The same energies for both HPC_60_ and qHPC_60_ fullerene phases are −1.372 eV and −1.281 eV, respectively. In the hPC_60_ fullerene phase, the C_60_ molecule is bonded by six bonds to its neighbors. In the qhPC_60_ phase, each fullerene is bonded to its neighbors through four single and two cycloaddition bonds. The atomic configurations and unit cells of optimized monolayer boron nitride and two polymerized sets of fullerenes in the HPC_60_ and qHPC_60_ form are depicted in Figure 1.

**Figure 2:**
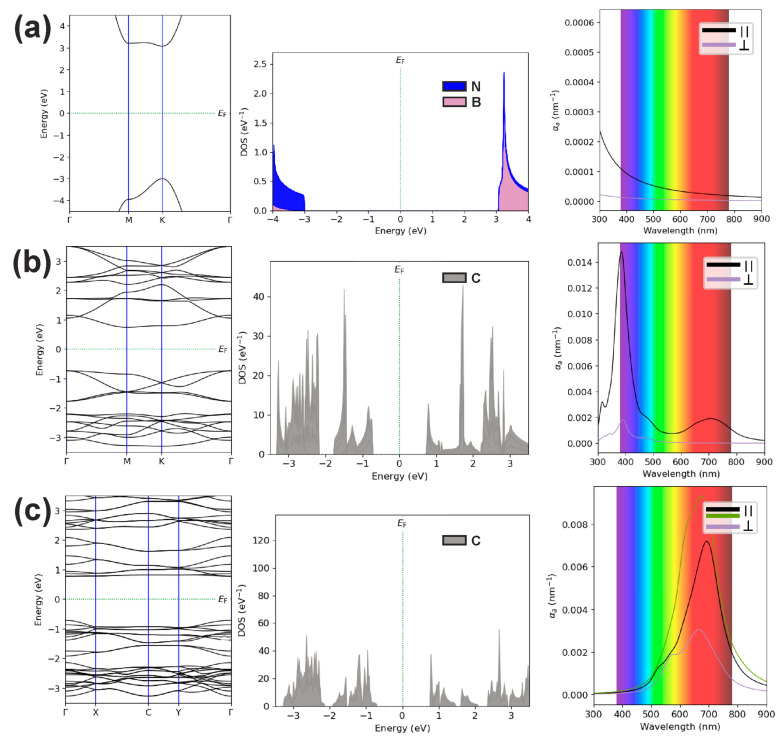
The electronic band structures, densities of electronic states, and optical absorption spectra of monolayer boron nitride (**a**), HPC_60_ fullerene phase (**b**), and qHPC_60_ fullerene phase (**c**).

Figure 1 shows relaxed atomic configurations and unit cells independent of each other, obtained in the course of solving the problem of optimization of geometric and energy parameters. In order to further compare the electronic and optical spectra of the assemblies and solitary layers, we present the results of calculations of electronic band structures (EBSs), densities of electronic states (DOSs), and optical absorption spectra (OAS) for all three layers separately. Figure 2 shows the EBS, DOS, and OAS of monolayer boron nitride, HPC_60_ fullerene phase, and qHPC_60_ fullerene phase. The characteristic values correspond to the DFT-HSE calculation scheme.

### 3.2. Geometric Structures and Properties of Heterostructures

**Figure 3:**
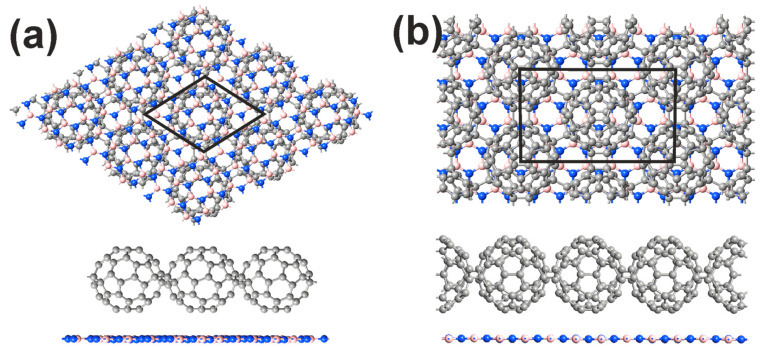
The atomic configurations and unit cells of optimized C_60_ polymer/*h*-BN heterostructures formed from monolayer boron nitride and HPC_60_ phase of polymerized fullerene (**a**) and monolayer boron nitride and qHPC_60_ phase of polymerized fullerene (**b**). Black lines highlight the unit cells of the heterostructures. Lattice parameter is 9.13 Å for the C_60_ (HPC_60_)/*h*-BN heterostructure; 15.4 Å and 8.91 Å for the C_60_ (qHPC_60_)/*h*-BN heterostructure.

An atomistic modeling approach makes it possible to easily construct nanoscale complexes from different materials into single assemblies and to draw reliable conclusions about the feasibility of synthesis. Moreover, these methods help to investigate the physicochemical properties and characteristics of predicted materials and structures. We constructed cells containing heterostructures of the “C_60_ polymer/*h*-BN” type, where fullerene HPC_60_ and qHPC_60_ phases acted as the polymers. Atomic images of these relaxed heterostructures are shown in Figure 3.

In the bilayer heterostructures under consideration, the lattices of the monolayer *h*-BN and both polymerized fullerene sets are incommensurate. For each case, we simulated a general “try-in” superlattice on the *h*-BN layer so that when superimposed on a C_60_ polymer unit cell, it would produce the closest commensurate configuration for the selected heterostructure. This is also performed when vertically assembling two different hexagonal layers of graphene and boron nitride. Since the C_60_ (HPC_60_)/*h*-BN heterostructure consists of two hexagonal atomic layers, by rotating one layer relative to the other, one can obtain a commensurate structural version with the lowest lattice mismatch. How to determine the twisting angle is shown in [25,26]. Using it, we found the most acceptable option with a small number of atoms in the supercell containing one fullerene and a rotated boron nitride layer with a rotated angle of 13.9⁰. The choice of the supercell in the case of the C_60_ (qHPC_60_)/*h*-BN heterostructure was carried out by selecting a rectangular boron nitride supercell, which was closest to the unit cell of the polymer C_60_ qHPC_60_ layer.

After DFT-PBE optimization, no strong distortions occurred in both the *h*-BN or polymerized C_60_ layers. As shown in Figure 3, no covalent bonds form between *h*-BN and HPC_60_ (qHPC_60_) fullerene phase. Apparently, the hetero-assembly is maintained due to van der Waals forces. The resulting (optimal) distance between the qHPC_60_ fullerene phase and *h*-BN is smaller compared to that of the C_60_ (HPC_60_)/*h*-BN heterostructure (3.03 Å vs. 3.25 Å). Accordingly, the bonding between *h*-BN and qHPC_60_ should be somewhat stronger. Let us check this by calculating the binding energy $E_{b}$ using the formula

(6) $$E_{b}=E_{\mathrm{hetero}}-(E_{\text{h-BN}}+E_{\mathrm{HPC}60/\mathrm{qHPC}60})$$

and take structural units (the monolayer and the set of same fullerenes in the polymerized form) as reference systems. Here, *E_hetero_* is the total energy of the cell containing the atomic configuration of the heterostructure composed of the polymerized fullerene phase and a substrate from a single-layer boron nitride; *E_h-BN,_ E_HPC60/qHPC60_* are the same, but for a free fullerene molecule and the polymerized fullerene phase, respectively. The binding energy of the C_60_ (qHPC_60_)/*h*-BN heterostructure is equal to –0.529 eV, while the binding energy of the C_60_ (HPC_60_)/*h*-BN heterostructure is –1.069 eV. Apparently, direct comparison between the bonding strength and interlayer distance is impossible in this case. The fullerene sets and *h*-BN are deformed differently in the resulting heterostructures. Values of the same B–N and C–C bond length in both individual species and corresponding heterostructures are presented in Table 1. As shown in Table 1, in both heterostructures, the B–N bonds are stretched. This stretching occurs due to the weak compressibility of fullerenes. The lengths of interfullerene bonds decrease more (1.59 Å vs. 1.55 Å) than other C–C bonds. In order to fit two structures into a single assembly and reduce mismatching, the weakest bonds will deform most easily. Apparently, it is interfullerene bonds that are such.

**Figure 4:**
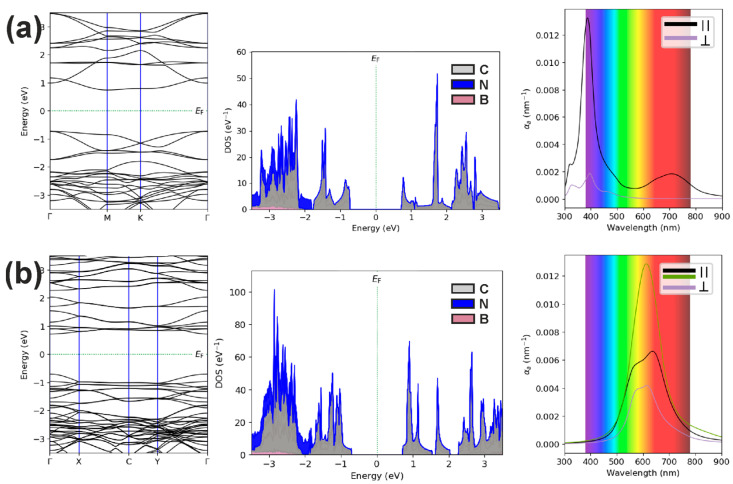
The electronic band structures, densities of electronic states, and optical absorption spectra of C_60_ polymer/*h*-BN heterostructures, where fullerene HPC_60_ (**a**) and qHPC_60_ (**b**) phases acted as the polymer.

Figure 4 shows the EBS, DOS, and OAS of C_60_ polymer/*h*-BN heterostructures, where fullerene HPC_60_ and qHP_C60_ phases acted as the polymer. As follows from the Figure 4, their spectra differ from the spectra of individual components of the heterostructures (Figure 2b,c). Moreover, Figure 4 indicates charge redistribution due to the presence of a boron nitride monolayer in the heterostructure. As follows from the calculated density of states, the states of nitrogen atoms make their contributions over a wide range of energies.

In the C_60_ (qHPC_60_)/*h*-BN heterostructure, small charge transfer occurs between the components with a redistribution of the electron density in them, which leads to a decrease in the band gap to 1.42 eV from 1.48 eV compared to the free fullerene qHPC_60_ phase. For the fullerene HPC_60_ phase, this difference is smaller: 0.02 eV. The ability to easily stretch the presented polymerized fullerenes greatly influenced the value of the band gap we obtained when using one or another atomistic calculation model. In this regard, the band gaps and light absorptions we obtained differed (by 5% and high transformation) from those listed in [5,6]. A summary of the calculated data on the structural and electronic properties of the considered heterostructures and individual layers is presented in Table 2. In the 3rd and 4th columns, the binding energies correspond to the formation of the sets from single fullerenes, but in the 5th and 6th columns, the binding energies correspond to the formation of the heterostructures.

The calculated optical absorption coefficients for components parallel and perpendicular to the heterostructure plane show strong anisotropic behavior, just as in the case of free C_60_ polymers. As can be seen, when using a monolayer boron nitride substrate for the HPC_60_ fullerene phase, the optical absorption is slightly weakened (0.0145 vs. 0.0132 nm^−1^), while for the qHPC_60_ fullerene phase, on the contrary, it increases (0.0128 vs. 0.0093 nm^−1^) and spectra change. In the obtained electronic spectra, strongly flattened minibands are visible near the conduction band minima and the valence band maxima, giving high DOS values at a number of certain energies. This indicates the possibility of observing resonant optical, nonlinear optical, and photoelectronic properties in the proposed heterostructures. Since the heterostructures considered do not have a center of symmetry and, therefore, are piezoelectric and non-linear optical media, they can be used to create new planar devices that use their nonlinear optical [27] and acoustoelectric [28] properties.

**Figure 5:**
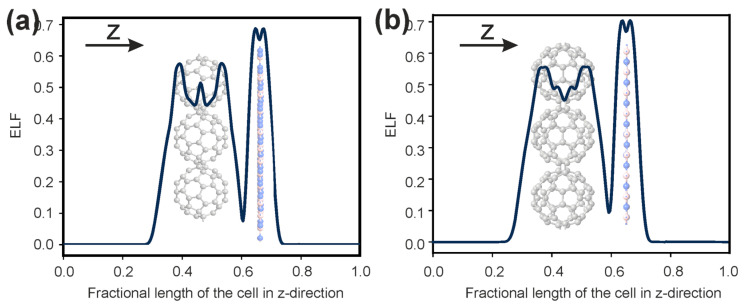
The ELF values projected onto line along z-direction of the constructed cells: (**a**) the case of C_60_ (HPC_60_)/*h*-BN heterostructure; (**b**) the case of C_60_ (qHPC_60_)/*h*-BN heterostructure.

Figure 5 shows the ELF values projected and summarized in a line along the z-direction of the constructed cells. Since the line “accumulates” all contributions along the preferred direction, the localization of electrons between the boron nitride monolayer and fullerene sets can be determined by the numerical value. The integrated ELF between both the fullerene sets and boron nitride monolayer does not exceed 0.1, which is very small.

## 4. Conclusions

In this study, we focused on the interaction of a boron nitride monolayer with polymerized fullerene molecules. The effect of interaction between a boron nitride substrate and a network of fullerenes, which is missing in the literature, was considered. The emerging van der Waals interaction, in some cases, changes the electronic and optical spectra. The calculated formation energies show that the deposition of fullerenes onto a single-layer boron nitride substrate is advantageous. The resulting heterostructure must be stable. Using the monolayer boron nitride dielectric substrate to support the presented fullerene sets is a good way to preserve their semiconducting properties. The choice of piezoelectric boron nitride substrate was dictated by interest in the considered van der Waals bilayers in mechanoelectric, photoelectronic, and electro-optical device applicability with the ability to control their properties.

## Figures and Tables

**Table 1:** Values of same bond length (Å) of the individual species and corresponding heterostructures. The values correspond to relaxed configurations. Selected bond lengths (A, D, C, and D) are presented in Figure S1 in Supplementary Materials. Interfullerene bonds are highlighted in bold.

Bond Type	*h*-BN	HPC_60_ Fullerene	qHPC_60_ Fullerene	C_60_ (HPC_60_)/ *h*-BN	C_60_ (qHPC_60_)/ *h*-BN
B–N	1.45			1.46	1.48
C–C		1.36 (A)	1.41 (A)	1.36	1.41
		1.40 (B)	1.45 (B)	1.39	1.45
		1.52 (C)	1.48 (C)	1.51	1.50
		**1.59 (D)**	**1.59 (D)**	**1.58**	**1.55**

**Table 2:** Values of lattice parameter *d*, binding energy *E_b_*, and band gap *E_g_* of the individual species and corresponding heterostructures.

	*h*-BN	HPC_60_ Fullerene	qHPC_60_ Fullerene	C_60_ (HPC_60_)/ *h*-BN	C_60_ (qHPC_60_)/ *h*-BN
*d*, Å	2.50	9.21	15.8; 9.17	9.13	15.4; 8.91
*E_b_*, eV		−1.372	−1.281	−1.069	−0.529
*E_g_*, eV	6.08	1.48	1.48	1.47	1.42

## Data Availability

Data are contained within the article.

## Supplementary Materials

The following supporting information can be downloaded at: https://www.mdpi.com/article/10.3390/polym16111580/s1, Figure S1: Selected bond lengths for analysis of tuning of fullerene sets in the C60 polymer/h-BN heterostructures (left—HPC60 fullerene set, right—qHPC60 fullerene set).
