# Supplementary material for: Bilayer C60 Polymer/h-BN Heterostructures: A DFT Study of Electronic and Optic Properties
Source: Polymers (Basel). 2024 Jun 3;16(11):1580. doi: 10.3390/polym16111580 (PMC11175054; doi:10.3390/polym16111580)
Supplement: Supplementary file 1 [file polymers-16-01580-s001.zip › polymers-2971963-supplementary.pdf]

Supporting Information

for paper:

"Bilayer C60 polymer/h-BN heterostructures: a DFT study of electronic and optic properties"

Authors:

Leonid A. Chernozatonskii[1,2], Aleksey I. Kochaev[3,4]

[1] Emanuel Institute of Biochemical Physics RAS, 4 Kosygin Street, 119334 Moscow, Russia
[2] Scientific School on Chemistry and Technology of Polymer Materials, Plekhanov Russian University of Economics, 117997 Moscow, Russia
[3] Research and Education Center "Silicon and Carbon Nanotechnologies", Ulyanovsk State University, 42 Leo Tolstoy Street, 432017 Ulyanovsk, Russia
[4]Laboratory of 2D nanomaterials in electronics, photonics and spintronics, National Research Nuclear University "MEPhI" 31 Kashirskoe sh., 115409 Moscow, Russia

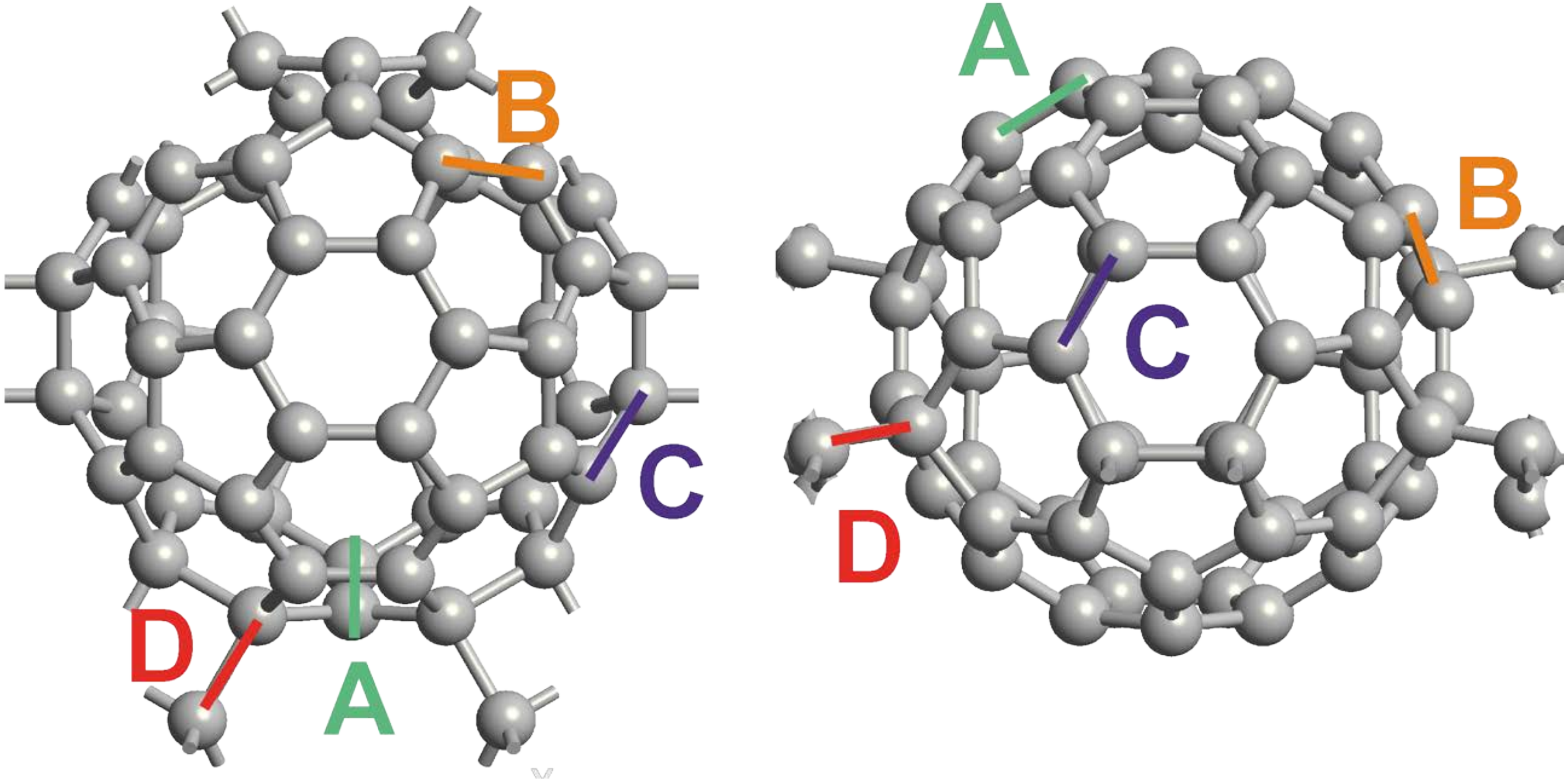

Figure S1. Selected bond lengths for analysis of tuning of fullerene sets in the C_{60} polymer/*h*-BN heterostructures (left - HPC60 fullerene set, right – qHPC60 fullerene set).
